# Context-dependent similarity searching for small molecular fragments

**DOI:** 10.1186/s13321-025-01032-1

**Published:** 2025-05-26

**Authors:** Atsushi Yoshimori, Jürgen Bajorath

**Affiliations:** 1 Institute for Theoretical Medicine, Inc., 26-1 Muraoka-Higashi 2-Chome, Fujisawa, Kanagawa 251-0012 Japan; 2 Department of Life Science Informatics and Data Science, B-IT, LIMES Program Unit Chemical Biology and Medicinal Chemistry, University of Bonn, Friedrich-Hirzebruch-Allee 5/6, 53115 Bonn, Germany; 3 Lamarr Institute for Machine Learning and Artificial Intelligence, University of Bonn, Friedrich-Hirzebruch-Allee 5/6, 53115 Bonn, Germany

**Keywords:** Molecular similarity, Context-dependent similarity, Small molecular fragments, Substituents, Similarity-property principle, Chemical similarity searching

## Abstract

Similarity searching is a mainstay in cheminformatics that is generally used to identify compounds with desired properties. For small molecular fragments, similarity calculations based on standard descriptors often have limited utility for establishing meaningful similarity relationships due to feature sparseness. As an alternative, we have adapted the concept of context-depending word pair similarity from natural language processing to evaluate similarity relationships between substituents (R-groups) taking latent characteristics into account. Context-dependent similarity assessment is based on vector embeddings as fragment representations generated using neural networks. With active analogue series as a model system to establish a global structure–activity context, we demonstrate that this approach is applicable to systematic similarity searching for substituents and increases the performance of standard descriptor representations. Context-dependent similarity searching is capable of detecting remote and functionally relevant similarity relationships between substituents. Alternative search queries are introduced focusing on individual substituents within a global substituent context or individual sequences of substituents establishing a local context. For similarity searching, different structural or structure–property contexts can be established, providing opportunities for various applications.

## Scientific Contribution

Previously, we introduced context-dependent similarity assessment for analogue series alignment. The approach is based on the concept of context-dependent similarity of words from natural language processing. Herein, the methodology is extended for similarity searching of small molecular fragments. Context-dependent similarity searching takes latent fragment features into account, representing a new approach for chemical similarity assessment.

## Introduction

Quantitative assessment of molecular similarity plays a central role in cheminformatics [1–3]. Similarity calculations can be carried out for the assessment of structural similarity for applications such as compound database retrieval or library design. However, similarity calculations are often not carried out to quantify chemical similarity of compounds per se, but rather as an indicator of similar molecular properties such as biological activity [4, 5]. The underlying concept is referred to as the similarity-property principle [4, 5]. Accordingly, similarity assessment is indispensable for the analysis of structure–activity relationships (SARs) in compound data sets [6, 7], which in turn provides a basis for the identification of leads and subsequent compound optimization [8–10]. In medicinal chemistry, analogues series (AS) resulting from compound optimization efforts represent a major source of SAR information [10, 11].

Molecular similarity calculations are typically carried out based on molecular descriptors such as fingerprints (bit string representations of a molecular structure or of its properties) or numerical physicochemical descriptors [1, 4, 5, 12, 13]. For the quantitative comparison of descriptor vectors, similarity metrics such as the Tanimoto coefficient [14, 15] are applied. A very popular approach in molecular similarity analysis is similarity searching where known reference molecules are used as templates to search databases for compounds having a similar activity (or other properties) [4, 5, 13, 14]. Similarity searching produces similarity-based rankings of database compounds (resulting from pairwise comparison of reference and database molecules), from which candidates are selected.

For small chemical structures or fragments, descriptor-based similarity values tend to insufficiently account for similarity relationships, due to sparse or narrowly distributed descriptor features, which also limits similarity searching in such cases [5, 12]. These limitations are difficult to address in conventional similarity calculations. Therefore, we have previously introduced an alternative approach to better account for similarity of small molecular fragments that is based on vector embeddings for the prediction of words in natural language processing [16, 17]. The resulting method for context-dependent similarity assessment was shown to quantify substituent similarity in a meaningful way, enabling the alignment of AS [18] for the detection of SAR transfer events [19, 20] and the identification of non-classical bioisosteric replacements [18]. In this work, we extend context-dependent similarity assessment and investigate whether the concept can be applied to fragment-based similarity searching. For small fragments, similarity searching using conventional descriptors has substantial limitations. Therefore, we use fragment vectors for similarity searching in a global context and also generate a new weighted embedding of fragment series, providing an additional local context. The inclusion of global and local contexts increases the information content of similarity searching for small fragments. We show that the new search methodology outperforms conventional approaches in similarity searching for small fragments such as substituents.

## Methods

### Context-dependent similarity

Similarity searching for small chemical structures such as fragments using conventional chemical descriptors is known to be difficult. For instance, fingerprint bit settings are typically very sparse for fragments, which often prevents the calculation of informative similarity values. For generating substituent-based alignments of AS, which requires a similarity matrix for substituents, we originally introduced the conventional fragment representation (CFR) metric to combine structural and property similarity for fragments, thereby attempting to increase the chemical information content of similarity calculations [12].

Subsequently, embedded fragment vectors (EFVs) adapted from natural language processing were generated using the Word2vec (W2V) neural network method for predicting words that are consistent with the context provided by neighboring words in a sentence [16]. Fragments in input sequences were represented as molecular strings. We showed that EFV-based calculation of substituent similarity was superior to CFR similarity for generating similarity matrices for AS alignments and identifying bioisosteric replacements of substituents [18]. In light of these findings, we have been interested in exploring whether EFV-based similarity calculations might also be applicable to similarity searching for substituents. For investigating proof-of-concept, as a context for similarity search calculations, we also considered AS representing sequences of substituents leading to increasingly potent compounds.

**Fig. 1:**
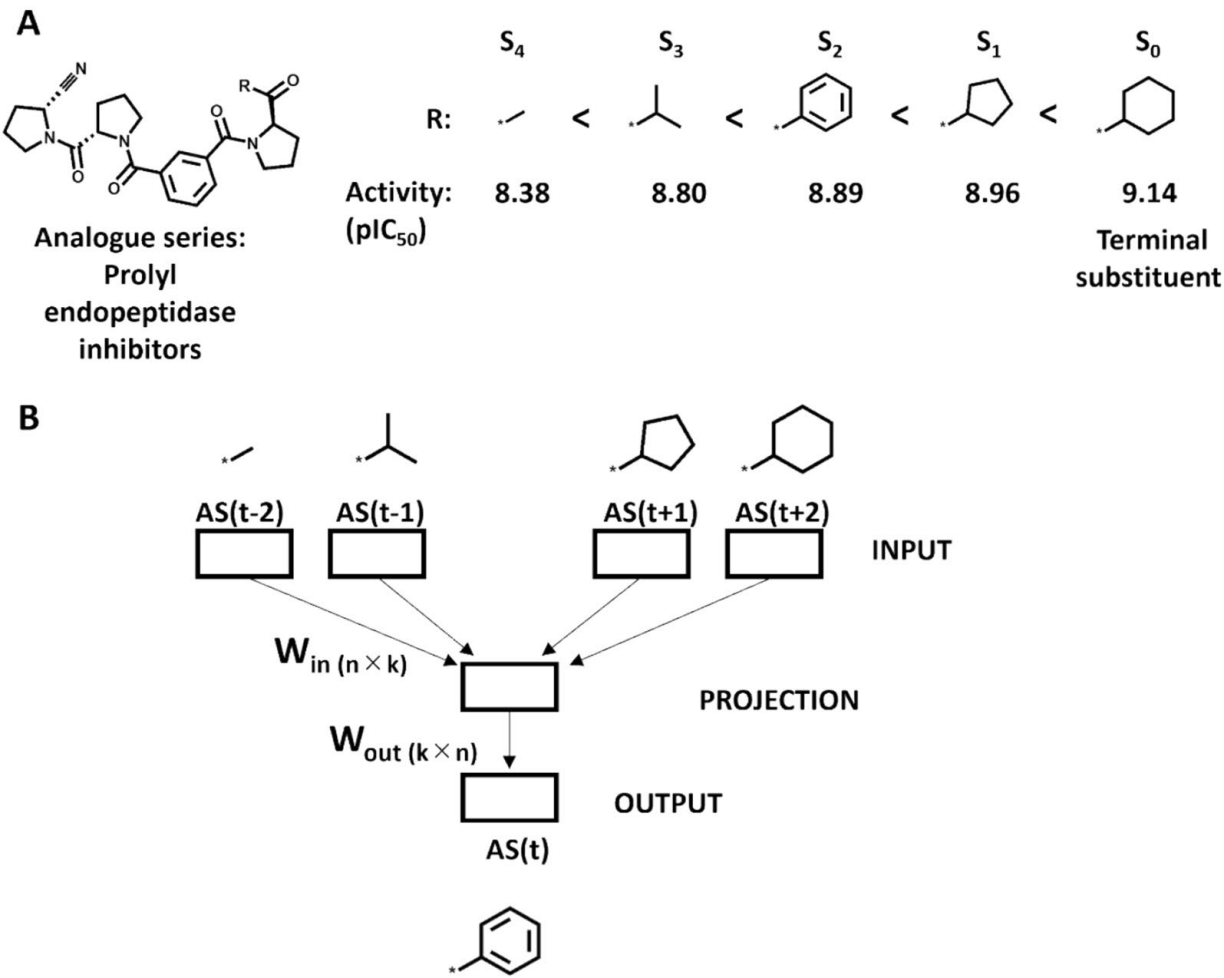
Generation of an embedded fragment vector. The construction of an EFV via W2V is illustrated. In (**A**), an exemplary training AS is shown, represented as a sequence of substituents ordered by increasing potency (<) of the analogues. S_x_ denotes the analogue/substituent that is X positions away from the terminal substituent (S_0_) having the highest potency (pIC_50_ value). **B** schematically illustrates the architecture of the CBOW_W2V model for EFV construction consisting of four input layers (with window size of 2), a projection, and an output layer. W_in_ and W_out_ are weights of the model and n accounts for the size of the vocabulary (corresponding to the different fragments in the AS) and k for the dimensionality of the EFV. Indices (t ± 1/2) define the position of an input substituent in an AS with respect to the prediction target at position t (here S_2_)

Figure 1A shows a potency-order AS represented as a sequence of substituents. Figure 1B schematically represents a W2V variant model (see below) consisting of a neural network with four input layers, a single projection layer, and an output layer. Here, a substituent is predicted based on the two preceding and following substituents in the AS. W_in(n×k)_ and W_out(k×n)_ are weights of the model, *n* is the number of unique substituents in the AS and *k* the dimensionality of the hidden layer (corresponding to the dimension of the EFV). Input fragments are represented as one-hot encoded vectors. The input of the projection layer consists of the mean vector of the individual one-hot encoded substituent vectors multiplied by W_in(n×k)_. A trained W2V variant model generates the EFV of each fragment from the weight W_in(n×k)_.

In the example in Fig. 1B, the two preceding and following substituents provide the AS-specific context for predicting the substituent S_2_ at position t in the AS using the W2V model.

### Concept of context-dependent similarity searching

We reasoned that EFV-based similarity assessment might be further extendable to and generalizable for similarity searching for small fragments. In similarity searching utilizing an AS context, we do not attempt to predict individual substituents at a given position of an AS, as illustrated in the example in Fig. 1B, but instead search for the terminal substituent S_0_ in an AS (representing the most potent compound). Therefore, different queries can be designed to search EFVs of the complete vocabulary (total number of unique substituents) generated during model training, as described above, and generate a Tanimoto similarity-based ranking of all EFVs relative to the query. The EFV of each substituent captures the context information provided by all AS it belongs to and defines its position in vector space. This is relevant for the selection or generation of similarity search queries (templates), as described in the following.

### Similarity search queries

To search for a terminal substituent S_0_ of an AS (Fig. 1A), any substituent in the AS such as S_1_, S_2_, S_3_, or S_4_ can be selected, given that EFVs derived during model training capture all sequential relationships between any pair of substituents found in an AS. Similarity searching based on the EFV of an individual substituent does not take other substituents in the AS into account and thus abstracts from the context of a given AS. The EFV is based on the global context established during training.

**Fig. 2:**
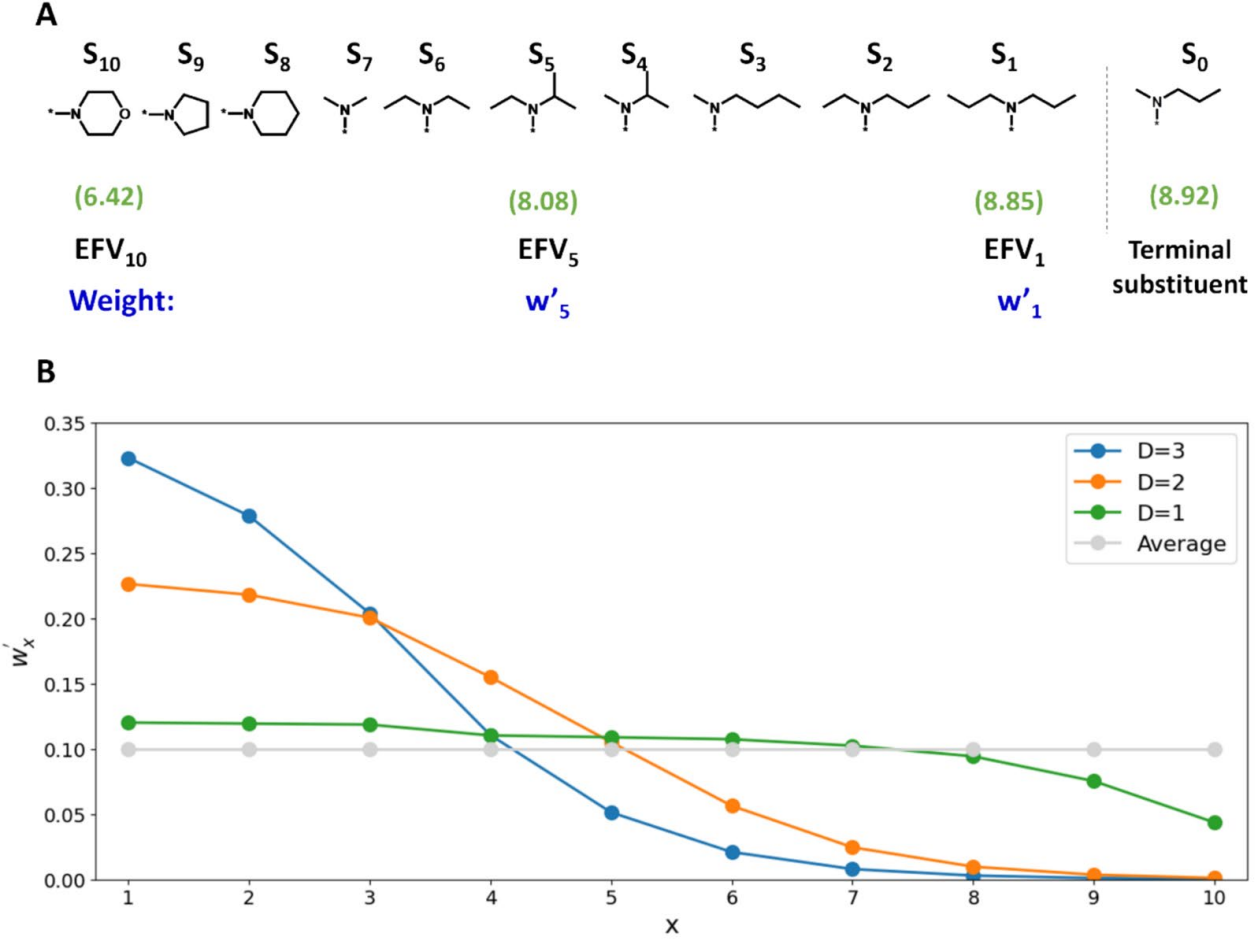
Substituent weights. **A** shows an exemplary AS with 11 substituents (S_x_) with selected pIC_50_ values (in parentheses). For this AS, **B** shows normalized weights (w.^’^_x_) of substituents 1–10 for different slope factors D and the average of all substituent weights (summing up to 1)

However, a formalism can also be derived to generate a query with contributions from each substituent of an AS, thereby emphasizing the local context of the AS (while retaining global context information in substituent EFVs). For this purpose, we generate the weighted average EFV (wEFV) of all substituents S_x_ (except S_0_) of an AS. Figure 2A shows an exemplary AS comprising 11 compounds, 10 of which are used to derive the query for S_0_.

The weight (w) for the EFV of S_x_ is defined as:

$${w}_{x}=\frac{{Pot}_{x}}{1+exp\left(x-\frac{L}{D}\right)}$$

Here, x denotes the position of a substituent in the AS, Pot_x_ is the potency (here pIC_50_ value) of the compound with substituent S_x_, L is number of substituents/analogues in the AS, and D a slope factor that can be arbitrarily set.

The normalized weight (w′_x_) for EFV of S_x_ is defined as:

$${w}_{x}{\prime}=\frac{{w}_{x}}{{\sum }_{x=1}^{L}{w}_{x}}$$

Finally, the weighted average wEFV of the AS is obtained as:

$$wEFV={\sum }_{x=1}^{L}{w}_{x}{\prime} {EFV}_{x}$$

Figure 2B reports normalized weights of substituents of the AS in Fig. 2A for different slope factors. For a slope factor of 1, weights remain close to the average (except for substituents most distant from S_0_). For increasing slope factors of 2 and 3, relative weights are close to 0 for most distant substituents and increase in a sigmoidal manner with decreasing distance of substituents to S_0_. Thus, relative weights of substituents in wEFVs of an AS can be adjusted through the use of alternative slope factors. Larger slope factors increase weight contributions from substituents that are close in sequence to the search target S_0_. wEFVs represent AS-based queries focusing similarity searching on the local context.

### Analogue series

For our analysis, a previously reported set of 113,113 AS with single substitution sites and activity against 2240 target proteins containing 26,795 unique substituents [18] was used. Compounds in all AS were ordered according to increasing potency. Hence, each AS represented an ascending compound potency gradient. This data set was extracted from ChEMBL [21] (release 29). Each AS exclusively consisted of compounds originating from the same publication with available IC_50_ values (standard relation “=”) for the same target and highest ChEMBL assay confidence score of 9 [21]. AS with at least three compounds were extracted from compound activity classes using the matched molecular pair (MMP) algorithm of Hussain and Rea [22] implemented in RDKit [23]. A value fragment (substituent) was permitted to consist of up to 12 non-hydrogen atoms and a maximum of 30% of the non-hydrogen atoms of the source compound.

### Fragment representations

Substituent fragments were represented using different descriptors including the Morgan fingerprint [24] and molecular quantum number (MQN) descriptors [25]. A folded version of the Morgan fingerprint with bond radius 2 and size of 1024 bits was generated with RDKit.

MQN descriptors include 42 atom, bond, or chemical group descriptors with different chemical characteristics [25]. They were calculated using RDKit and recorded in a vector format. The combination of the Morgan FP and MQN descriptors provided the CFR representation. As an alternative to conventional descriptors, EFVs were generated for substituents using the W2V neural network method for predicting words that are consistent with the context provided by neighboring words in a sentence [16]. In our fragment-based application of W2V, substituents correspond to words and sequences of substituents representing AS correspond to sentences. W2V uses large numbers of words or sentences as input to generate high-dimensional real-valued vector representations of words such that similar words are close to each other in vector space [16]. This feature provides the basis for context-dependent similarity calculations. The continuous bag of words (CBOW) variant of W2V (CBOW_W2V) [26] is capable of generating an EFV for a given target fragment based on queries combining adjacent substituents in an AS, as demonstrated previously [18]. For our current study, CBOW_W2V models were generated using Gensim [27]. For its word2vec.Word2Vec function, the following input parameter settings were used: vector_size: 100; window: 5; min_count: 1; sg: 0; seed: 8; workers: 1.

### Fragment similarity

Morgan fingerprint similarity of substituents was calculated using the Tanimoto coefficient [15] and MQN similarity (${S}_{ij}^{MQN}$) was calculated as [25]:

$${S}_{ij}^{MQN}=\frac{1}{\left(1+\frac{1}{42}{\sum }_{l=1}^{42}\left|{MQN}_{l}^{i}-{MQN}_{l}^{j}\right|\right)}$$

where ${MQN}^{i}$ and ${MQN}^{j}$ represent MQN descriptors of fragment *i* and *j*, respectively.

CFR similarity was then calculated by averaging Tanimoto and MQN similarity [12]. For the Tanimoto coefficient and MQN similarity measures, the maximal value is 1.

In our original EFV implementation, context-dependent similarity was quantified using cosine similarity [18], which is typically used for comparing word vector embeddings. For context-dependent similarity searching and direct comparison with standard similarity search methods, EFV similarity was quantified using the Tanimoto coefficient for real-valued vectors [15]. Accordingly, for a query substituent, rankings of substituents were generated based on Tanimoto similarity of their EFVs.

### Embedded fragment vectors for similarity searching

Five independent CBOW_W2V models were derived to generate EFVs for context-dependent similarity searching based on sequences of substituents represented as canonical SMILES strings [28]. In each case, 90% of the 113,113 AS were selected for model training. From the remaining 10%, 1000 AS consisting of more than eight compounds were randomly selected as test sets for similarity searching. The training sets contained on average 25,751 unique substituents, defining the vocabulary for model derivation. All substituents from test AS were also required to be contained in vocabulary list of the training data. Test AS were used to predict the terminal (most potent) substituent/compound of each AS by similarity searching based on different queries, as specified below. CBOW_W2V models were trained based on complete AS (sentences) representing sequences of substituents (words), providing context information for individual substituents (across many AS). From these input data, EFVs for individual substituents were generated during model training.

For assessing similarity search calculations, we determined ranks of correctly detected substituent fragments within the first bin of the rankings, that is, the top-50 positions, which was rationalized as follows. The training sets contained on average 25,751 unique substituents. Thus, substituents in the first bin corresponded to less than 0.2% of all substituents. It follows that ranks within the top-50 were indicative of a strong statistical enrichment of correct fragments. Moreover, evaluating on the order of 50 substituents for candidate compounds based on similarity searching is, in our experience, common practice in medicinal chemistry.

## Results and discussion

### Similarity searching using substituent queries

**Fig. 3:**
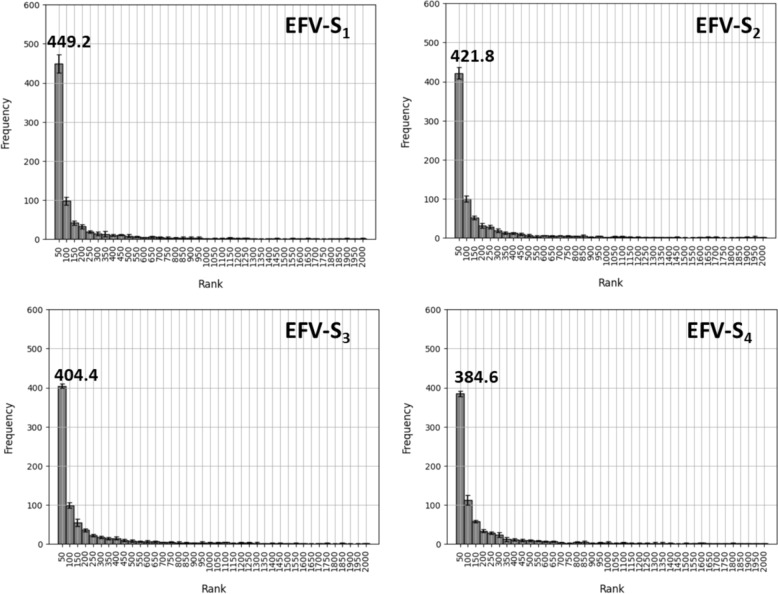
Similarity search for a terminal substituent S_0_ using S_x_ as a query. For the test data set, similarity searches for the terminal substituent S_0_ were carried out using EFVs of S_1_, S_2_, S_3_, or S_4_ as input, respectively. EFV-S_x_ indicates that the EFV was generated for substituent S_x_. The corresponding histogram reports the frequency of the terminal substituent across different similarity search rank intervals (as the mean with standard deviation over five search trials with EFVs from independent models). Mean frequency is reported above the first rank interval (1–50)

Initially, we systematically searched for the terminal substituents of 1000 test AS using EFV queries of substituents S_1_, S_2_, S_3_, or S_4_, respectively, from five independently derived CBOW_W2V models (see “Methods”). In each case, more than 25,000 substituents were ranked based on Tanimoto similarity of the EFV vectors (pairwise comparisons). Figure 3 shows the distribution of the terminal substituents of the 1000 test AS over different rank intervals for the four substituent queries over five independent search trials (using their EFVs from different models).

The search calculations revealed a high enrichment of many terminal substituents at top ranks. For S_1_-S_3_, an average of more than 400 terminal substituents was detected at rank 1–50 (with a maximum of ~ 449 substituents for S_1_), an encouraging finding, providing initial proof-of-concept for context-dependent similarity searching. There was a decrease in the average number of highly ranked substituents with increasing distance of S_x_ to S_0_ (to a mean of ~ 384 substituents for S_4_), resulting from gradually decreasing context-dependent similarity of the query substituent to S_0_. Notably, this position- and distance-dependent decrease was not caused by the specific (local) AS context, given that these substituents were used as individual queries, but resulted from positional preferences of substituents in the global AS context established during training. In other words, substituents that frequently occurred close to each other in AS representing compound optimization paths occupied proximal positions in EFV vector space.

**Fig. 4:**
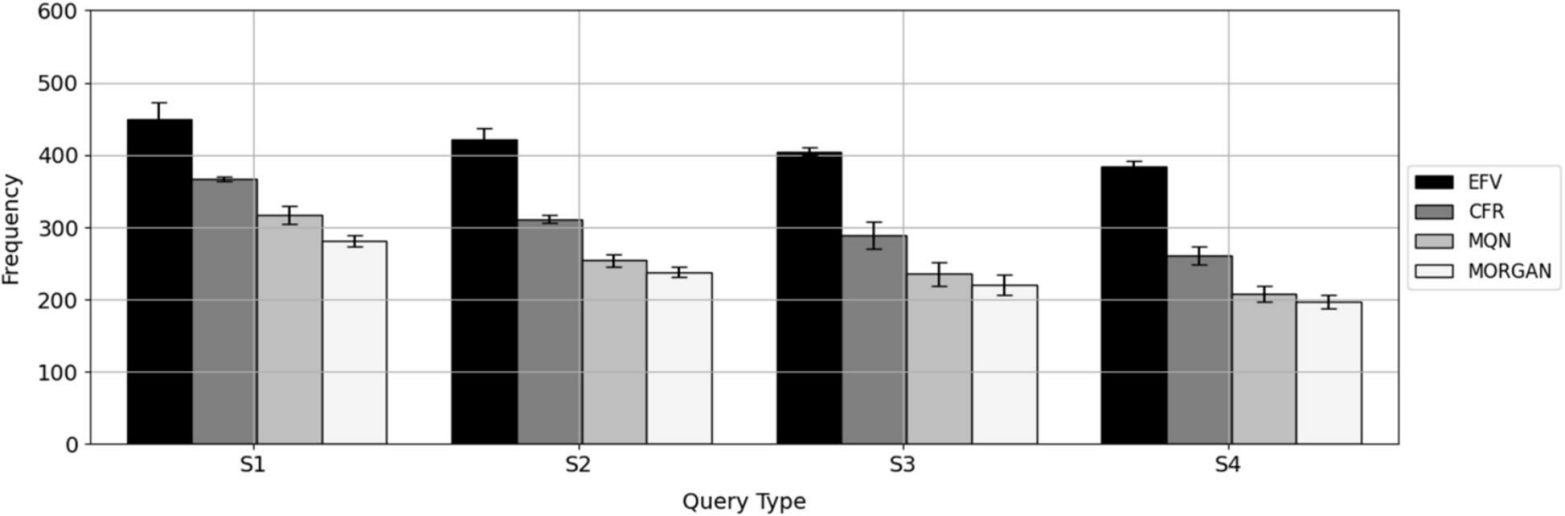
Similarity search comparison. For the test data set, histograms report the mean frequency (with standard deviation over five trials) of the terminal substituents in the first rank interval (top 50) for similarity searches using alternative representations (Query Type) of the S_1_–S_4_ queries including EFV, CFR, MQN, and the Morgan fingerprint (MORGAN)

Figure 4 compares the frequency of terminal substituents in the first rank interval obtained with the substituent EFVs with CFR, MQN, and MORGAN control calculations.

**Fig. 5:**
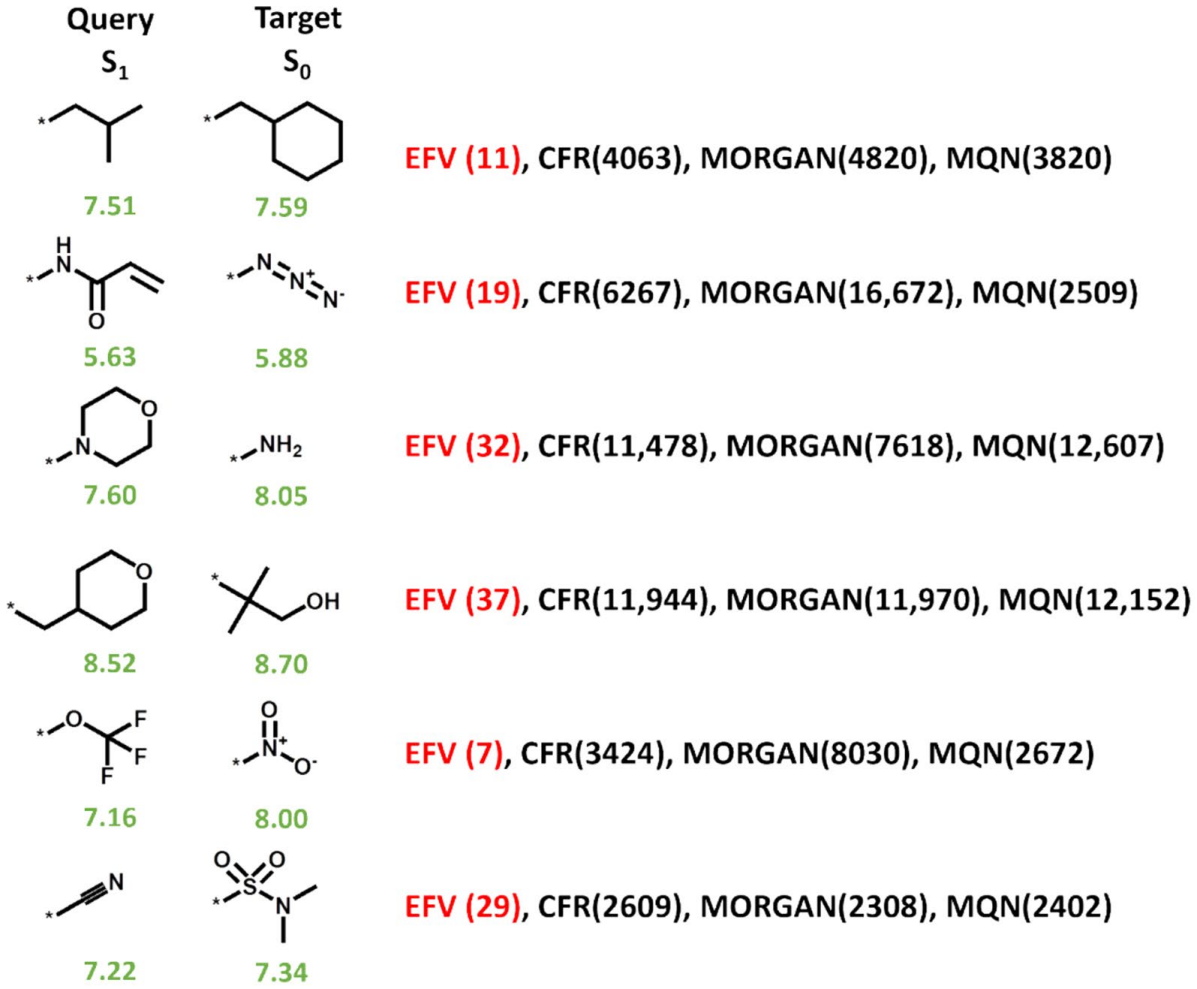
Exemplary search results. Similarity search examples are shown using S_1_ as a query for terminal substituents S_0_ using EFV, CFR, MQN, and MORGAN similarity calculations. Values in parentheses report rank positions and values beneath S_1_ and S_0_ fragments represent pIC_50_ values of the corresponding analogues. These examples are characterized by the presence of remote structural similarity between the query (S_1_) and target (S_0_) substituents

For S_1_, S_2_, S_3_, and S_4_, substituent, EFVs produced on average ~ 100 more highly ranked terminal substituents than CFR similarity calculations, followed by MQN and Morgan fingerprint calculations. Furthermore, especially for the S_1_ query, differences in rank positions between EFVs and similarity control calculations were often exceedingly large. Figure 5 shows representative examples of terminal substituents that were highly ranked based on EFVs, but had low ranks between ~ 2400 and ~ 12,600 in similarity control calculations.

In these cases, structural similarity between the query and target fragments was typically remote (or even non-obvious), although these substituents had a tendency to co-occur in AS representing compound optimization paths, thus highlighting the influence of the global context on EFV-based similarity calculations. By contrast, in standard descriptor-based similarity calculations, low values and ranks were obtained. As an additional control, Morgan FP Tanimoto similarity calculations were repeated using the more compact and simpler standard version of MACCS structural keys. While rank variations were observed for these FPs, as is usually the case, MACCS-based ranks were also consistently low and mostly comparable in magnitude to Morgan FP. Overall, the search performance was comparable and no general FP-dependent advantages were observed. Taken together, the findings revealed that EFVs were generally superior to descriptor-based approaches in these calculations. EFVs combined structural similarity and latent property similarity, in this case, similar potency within an AS-based gradient. This caused a tendency for substituents to co-occur in AS, given that similar substituents often resulted in similar compound potency, in accord with the similarity-property principle. The introduction of the CFR metric also aimed at combining structural and chemical property similarity for the comparison of substituents, as discussed above, albeit in a context-independent manner, and was less accurate in ranking terminal substituents.

### Similarity searching using analogue series-based queries

**Fig. 6:**
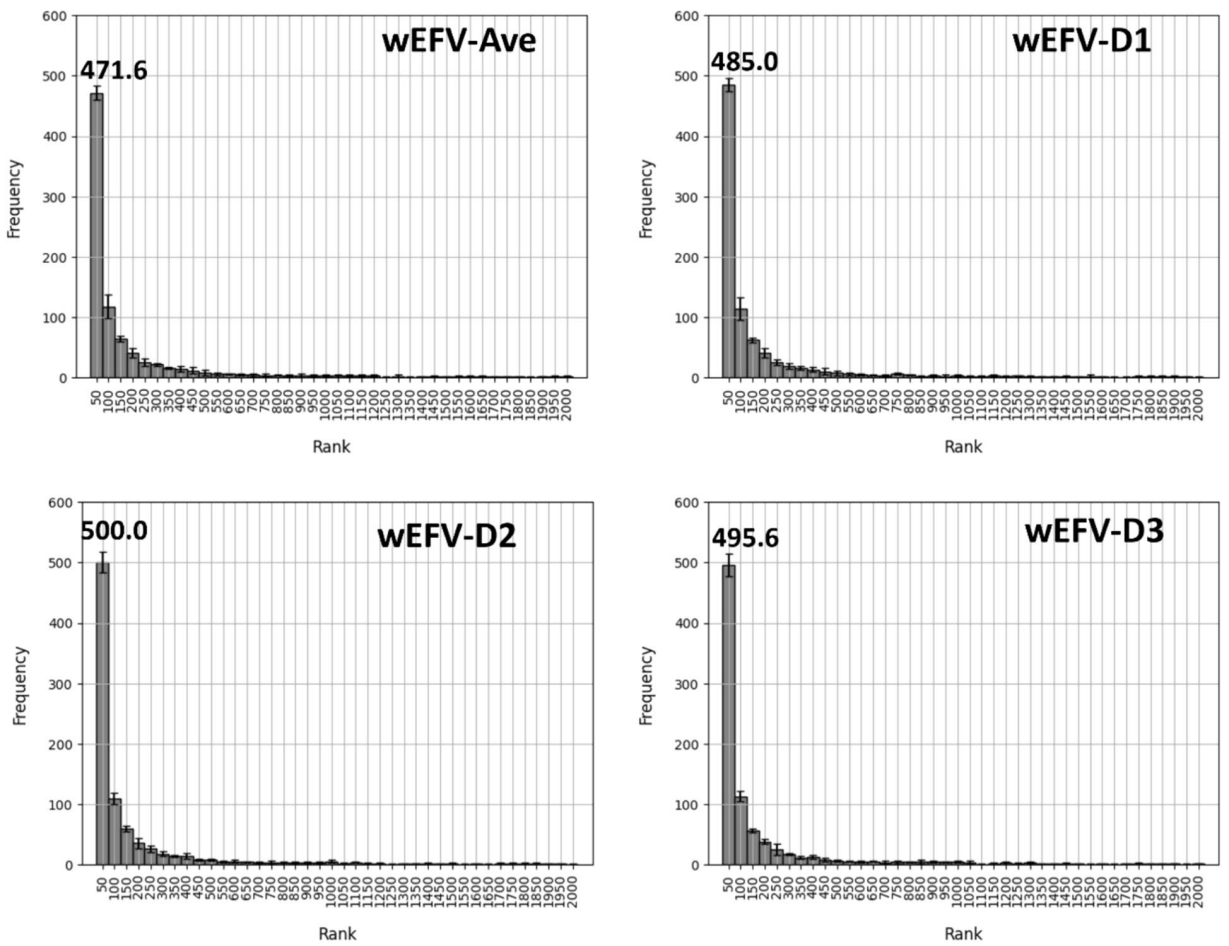
Similarity search for a terminal substituent using weighted EFVs. For the test data set, similarity searches for the terminal substituent S_0_ were carried out using wEFVs with different slope factors (D = 1–3) or the average (Ave) with constant weights. wEFV queries are composed of all substituents of an AS with position-specific weights. The histogram reports the frequency of the terminal substituent across different rank intervals (as the mean with standard deviation over five trials). The mean frequency is reported above the first rank interval (1–50)

We then repeated the context-dependent search calculations with different AS-based wEFV queries emphasizing the local AS context. Figure 6 shows the distribution of the terminal substituents of the 1000 test AS over different rank intervals for wEFV queries with different slope factors (or the average substituent weight) over five independent search trials.

For the average wEFV with constant weights, serving as a wEFV baseline, an increase to a mean of 471.6 terminal substituents in the first rank interval was observed compared to the best-performing substituent query (EFV-S_1_) in Fig. 3. Using wEFVs with slope factors further increased the search performance to a mean of 485.0 (wFV-D1), 500.0 (D2), and 495.6 (D3) terminal substituents in the first rank interval. Thus, the use of wEFVs with distance-dependent weights relative to the target substituent yielded overall best rankings, with closely comparable results for slope factors of 2 and 3.

**Fig. 7:**
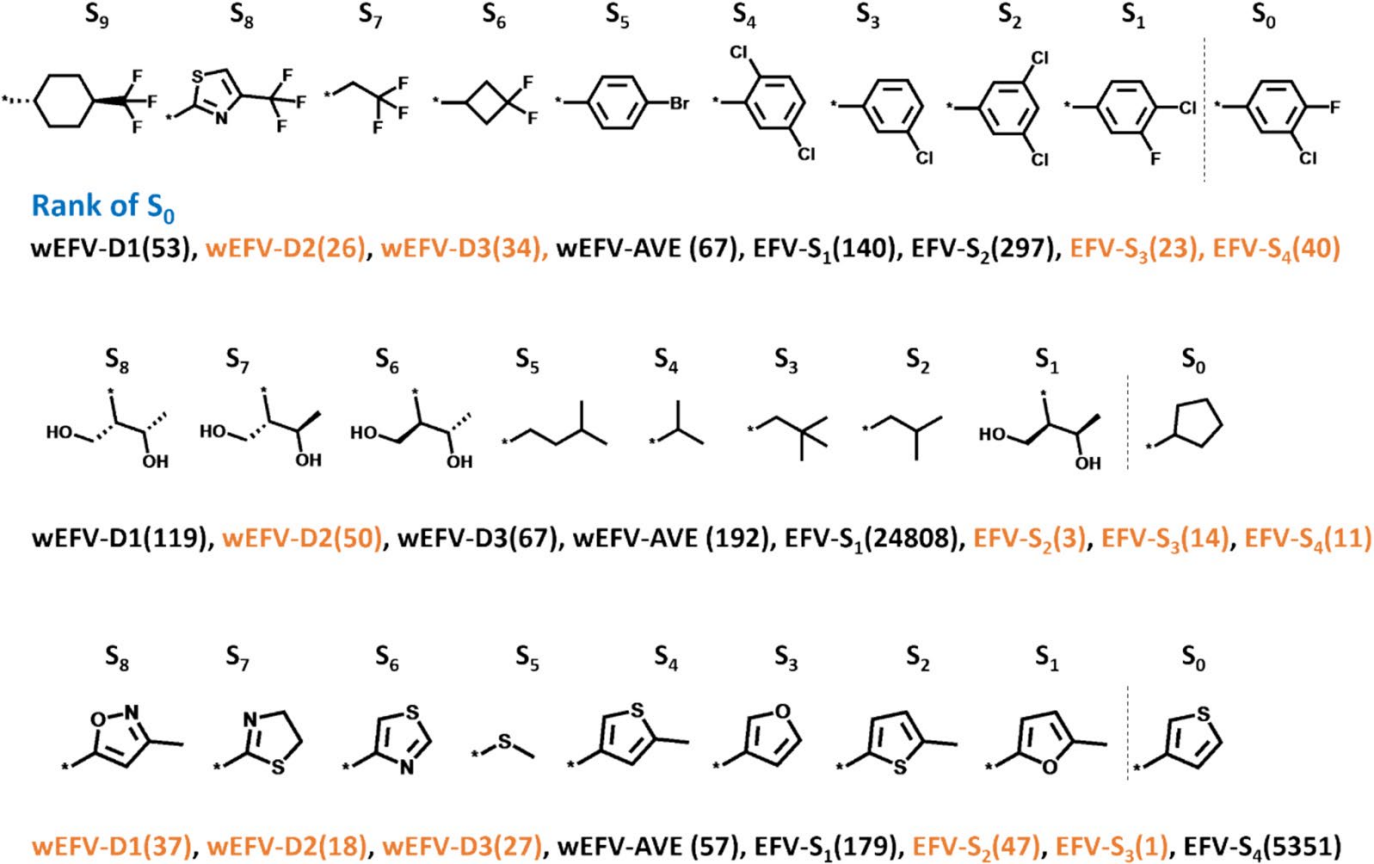
Exemplary search results for substituent-dependent and weighted EFVs. For three exemplary AS, results of similarity searches are shown using wEFV-D1, wEFV-D2, wEFV-D3, wEFV-AVE, EFV-S_1_, EFV-S_2_, EFV-S_3_, EFV-S_4_ as alternative query representations. For each query, the rank of the terminal substituent S_0_ is reported in parentheses. EFV variants yielding S_0_ ranks of 1–50 are shown in orange

Figure 7 compares search results using different substituent EFVs and weighted EFVs for exemplary AS.

In all three AS, substituents were for the most part structurally related. For instance, in the AS at the top of Fig. 7, S1, S2, and S3 were close analogues of the terminal substituent. EFV-S_3_ yielded the highest rank of the terminal substituent (rank 23), followed by wEFv-D2 and -D3 (rank 26 and 34, respectively). Hence, in this case, the structural relationships were well accounted for by substituent- or AS-based wEFV queries. In the AS in the middle of Fig. 7, S_1_ and S_0_ were structurally distinct, different from other adjacent substituents. Here, S_1_ was structurally most similar to its stereoisomers S_6_, S_7_, and S_8_. In this case, substituent queries for S2, and S3, and S4, which were more similar to S_0_ than S_1_, yielded high ranks for S_0_ (rank 3 based on EFV-S_2_), followed by wEFV-D2 (rank 50), hence prioritizing the global context for this AS of rather unusual composition. Moreover, for the AS at the bottom of Fig. 7, having structurally related substituents, two EFV and three wEFV queries produced high ranks of the terminal substituent (rank 1 for EFV-S_3_). Taken together, these examples illustrate that EFV and wEFV queries often produced similar results, despite the overall slightly higher search (rank) performance of wEFV queries, as revealed in Figs. 3 and 6.

## Conclusions and outlook

In this study, we have addressed the task of similarity searching for small molecular fragments based on the concept of context-dependent similarity that we previously adapted for the alignment of AS and for the analysis of SAR transfer events. Context-dependent similarity assessment originates from natural language processing where it is used to detect word pair relationships in high-dimensional vector spaces and predict words in sentences. In our adaption, sequences of substituents from AS were used to establish a molecular context for systematic similarity searching for individual substituents. Notably, establishing a context for similarity searching makes it possible to take latent characteristics of compounds or fragments into account, which directly relates to the similarity-property principle and a major goal of chemical similarity searching, that is, the identification of molecules having similar properties of interest. As a consequence, context-dependent similarity searching enables the detection of remote and property-oriented similarity relationships between molecular fragments that are difficult to capture based on conventional decriptor representations, as demonstrated herein. In our proof-of-concept study, a fragment-activity context was established for substituent similarity searching based on AS. However, different contexts can be generated. For instance, substituents can be ordered in (distinct and overlapping) sequences according to properties such as hydrophobicity and increasing molecular mass, hydrophilic character with increasing polarity, aromatic or non-aromatic ring systems with increasing heteroatom content etc. to provide alternative contexts for model derivation. Depending on their design, such contexts might put more emphasis on chemical characteristics or properties/functional features, and different latent properties can be considered. Once EFVs are obtained, similarity searching in vector space can be generalized by selecting any EFVs as queries. Another attractive feature of context-dependent similarity searching is the ability to consider alternative queries for search calculations such as EFVs to preferentially emphasize a global context or wEFVs to focus on a local context provided by a compound series or subset (essentially corresponding to the use of multiple reference compounds in conventional similarity searching). Thus, search calculations in fragment-property contexts can be tuned in various ways, depending on the specific search tasks. In light of our findings, context-dependent similarity searching for small molecular fragments is versatile and further expands the spectrum of similarity search approaches in cheminformatics.

## Data Availability

All data and source code are available via the following link: https://uni-bonn.sciebo.de/s/Yapj29frhJZ0pdK.

## Abbreviations

AS: Analogues series
CBOW: Continuous bag of words
CFR: Conventional fragment representation
EFV: Embedded fragment vector
FP: Fingerprint
MMP: Matched molecular pair
MQN: Molecular quantum number
SAR: Structure–activity relationship
W2V: Word2vec
